# Genetic Variants of *LDLR* and *PCSK9* Associated with Variations in Response to Antihypercholesterolemic Effects of Armolipid Plus with Berberine

**DOI:** 10.1371/journal.pone.0150785

**Published:** 2016-03-25

**Authors:** Isabel De Castro-Orós, Rosa Solà, Rosa María Valls, Angel Brea, Pilar Mozas, Jose Puzo, Miguel Pocoví

**Affiliations:** 1 Departamento de Bioquímica y Biología Molecular y Celular, Universidad de Zaragoza, Instituto de Investigación Sanitaria Aragón (IIS Aragón), Zaragoza, Spain; 2 Unitat de Recerca de Lipids i Arteriosclerosi, CIBERDEM, Servei de Medicina Interna, Hospital Universitari San Joan, IISPV Facultat de Medicina, Universitat Rovira i Virgili, Reus, Spain; 3 Unidad de Lípidos, Servicio de Medicina Interna, Hospital Universitario San Pedro, Logroño, Spain; 4 Servicio de Bioquímica Clínica. Hospital Universitario Miguel Servet, Zaragoza, Spain; Centro Cardiologico Monzino, ITALY

## Abstract

**Background:**

Armolipid Plus (AP) is a nutraceutical that contains policosanol, fermented rice with red yeast, berberine, coenzyme Q10, folic acid, and astaxanthin. It has been shown to be effective in reducing plasma LDL cholesterol (LDLc) levels. In the multicenter randomized trial NCT01562080, there was large interindividual variability in the plasma LDLc response to AP supplementation. We hypothesized that the variability in LDLc response to AP supplementation may be linked to *LDLR* and *PCSK9* polymorphisms.

**Material and Methods:**

We sequenced the *LDLR* 3′ and 5′ untranslated regions (UTR) and the *PCSK9* 5′ UTR of 102 participants with moderate hypercholesterolemia in trial NCT01562080. In this trial, 50 individuals were treated with AP supplementation and the rest with placebo.

**Results:**

Multiple linear regression analysis, using the response of LDLc levels to AP as the dependent variable, revealed that polymorphisms rs2149041 (c.-3383C>G) in the *PCSK9* 5′ UTR and rs14158 (c.*52G>A) in the *LDLR* 3′ UTR explained 14.1% and 6.4%, respectively, of the variability after adjusting for gender, age, and BMI of individuals. Combining polymorphisms rs2149041 and rs14158 explained 20.5% of this variability (*p* < 0.004).

**Conclusions:**

Three polymorphisms in the 3′ UTR region of *LDLR*, c.*52G>A, c.*504G>A, and c.*773A>G, and two at the 5′ UTR region of *PCSK9*, c.−3383C>G and c.−2063A>G, were associated with response to AP. These results could explain the variability observed in the response to berberine among people with moderate hypercholesterolemia, and they may be useful in identifying patients who could potentially benefit from supplementation with AP.

## Introduction

Hypercholesterolemia is a major risk factor for coronary heart disease. Several studies have demonstrated that lowering elevated plasma total cholesterol (TC) levels, particularly low-density lipoprotein cholesterol (LDLc), is beneficial for patients with borderline to mildly elevated TC and LDLc levels [1–4].

There is growing interest in using nutraceuticals for hypercholesterolemia management for two reasons: (1) Patients with metabolic conditions that can be addressed by nutraceuticals may prefer therapeutic management that does not involve drug treatment. (2) There may be a high incidence of clinical side effects associated with the drugs used to treat their condition [5]. This is particularly the case for patients with hypercholesterolemia who are taking 3-hydroxy-3-methylglutaryl-coenzyme A (HMG-CoA) reductase inhibitors, also known as statins. Statins are effective at reducing plasma cholesterol and the risk of cardiovascular disease (CVD). Although they have proven efficacy, they are associated with a relatively high incidence of clinical side effects, such as myopathy and myalgia [6–8].

The nutraceutical Armolipid Plus (AP) was recently reported to be associated with significant improvements in plasma lipids, insulin resistance, and other components of metabolic syndrome; it was associated with an overall decrease in the risk of CVD in a population with hyperlipidemia and medium-to-high CVD risk [9–13]. AP contains policosanol, fermented rice with red yeast, berberine, coenzyme Q10, folic acid, and astaxanthin. It has been shown that fermented rice with red yeast contains lovastatin, and its hypocholesterolemic effects were described in a meta-analysis [14]. Policosanols and fermented rice with red yeast have a hypocholesterolemic effect by inhibiting cholesterol through regulation of overexpression of HMGCoA reductase enzymatic activity [15] and also by decreasing HMG-CoA reductase activity by activating AMP-kinase [16].

Berberine has a hypocholesterolemic effect distinct from that of the statins [16]. The mechanism involves post-transcriptional upregulation of LDL receptor (LDLR) through mRNA stabilization. Its action is independent of sterol regulatory binding proteins (SREBP), but it is dependent on extracellular signal–regulated protein kinase (ERK) activation [17]. The mRNA stabilization mediated by berberine involves the 5′ proximal section of the 3′ untranslated region (UTR) of *LDLR* [17]. Moreover, berberine downregulates proprotein convertase subtilisin/kexin type 9 (PCSK9). It was recently reported that berberine also exerts inhibitory effects on the expression of PCSK9 protein and mRNA in HepG2 cells [18]. *In vitro*, BBR inhibits PCSK9 mRNA and protein expression through the *SRE* and *HNF1* promoter sites [19]. It has been shown that the inhibitory effect of berberine is partially abolished by single mutations in the SRE or HNF1 binding sites of *PCSK9* [19].

A recent study found considerable interindividual variation in the response of plasma LDLc levels to AP supplementation [13]. This variation may be caused by genetic differences, but the specific genes involved in the hypocholesterolemic effects of AP are largely unknown. We hypothesized that the variability in plasma lipid responses to AP supplementation could be linked to interactions of berberine with the *PCSK9* and *LDLR* promoters, as well as with *LDLR* 3′ UTR variants, because such interactions could potentially alter the transcriptional activity and mRNA stabilization of both genes. The aim of the present study was to determine if genetic variations in *PCSK9* and *LDLR* were associated with differences in individual hypocholesterolemic responses to the nutraceutical AP.

## Materials and Methods

### Study subjects

The subjects of the present study were participants in a randomized, double-blind, parallel, controlled, and multicenter trial (ClinicalTrials.gov number NCT01562080) conducted in hypercholesterolemic subjects with low CVD risk. The participants received one tablet/day of AP (*n* = 51, mean age ± SD 49.91 ± 11.61 years) or placebo (microcrystalline cellulose; *n* = 51, mean age 52.37 ± 11.15 years) for 12 weeks with dietary recommendations. The AP tablets contained berberine (500 mg), red yeast rice extract (200 mg), policosanol (10 mg), folic acid (0.2 mg), coenzyme Q_10_ (2 mg), and asthaxantine (0.5 mg).

The subjects’ characteristics and clinical trial details were described previously [13]. Briefly, the study included a total of 102 participants with low CVD risk and mild-to-moderately elevated LDLc (130–189 mg/dL) without hypolipemic therapy. Among the exclusion criteria were any concomitant chronic disease, triglycerides (TG) > 3.97 mmol/L, pregnancy or lactation, and history of CVD. At 12 weeks, compared to placebo, AP reduced LDLc by 26.9%, apolipoprotein (Apo) B-100 by 26.6%, total cholesterol/HDLc ratio by 25.5%, and ApoB/ApoA1 ratio by 28.6%, while ApoA1 was increased by 2.5% (*p* < 0.05) [13].

All subjects provided written informed consent to a protocol approved by the ethical review boards of Hospital Virgen del Rocío (Sevilla), Hospital San Jorge (Huesca), Hospital San Pedro (Logroño), Hospital Gregorio Marañón (Madrid), Hospital la Fe (Valencia) and Hospital San Joan (Reus). Protocols were in accordance with the Helsinki Declaration and good clinical practice guidelines of the International Conference of Harmonization (ICH GCP), and the randomized trial was conducted in accordance with the extended CONSORT 2010 guidelines.

### Genetic analysis

Genomic DNA was extracted from peripheral blood leukocytes with the DNA Extraction G BACC3 Nucleon kit (General Electric). Using TRANSFACT®, MAPPER, and JASPAR, single nucleotide variants (SNVs) in potential binding sites for transcription factors in the *LDLR* and *PCSK9* 5′ UTR were selected according to their likelihood of having a minor allele frequency of >5% according to European data from the 1000 Genomes Project. SNVs previously analyzed by our group were also included [20]. The SNVs selected in these regions were: rs2149041, rs2479406, rs17111503, rs2479408, and rs2479409 in *PCSK9* and rs17242346, rs17248720, and rs17249120 in *LDLR*. To genotype these SNVs, two fragments (from c.−2126 to c.−1287 and from c.−883 to c.−637) and three fragments (from c.−3472 to c.−2752, from c.−2210 to c.−1937, and from c.−1394 to c.−738) were amplified from *LDLR and PCSK9* 5′ UTR, respectively. In addition, a 1008 bp *LDLR* fragment from c.* 18 to c.*1026 was amplified. All primers are listed in S2 Table. The obtained amplicons were purified with ExoSTAR (GEHealthcare) and sequenced in both 5′ and 3′ directions in an ABI 3500xl DNA analyzer (Applied Biosystems).

### Statistical analysis

All statistical analyses were performed with SPSS software v.20 (SPSS Inc.). Data are presented as mean ± standard deviation (SD) for continuous variables, as median and interquartile range for variables with a skewed distribution, and as frequency for categorical variables. The 51 subjects who took AP were classified according to the detected SNVs in the *PCSK9* 5′ UTR and *LDLR* 3′ and 5′ UTR regions, and statistical analyses of interindividual variations in LDLc and total cholesterol were performed. Student’s *t-*test and Mann-Whitney U test were used as appropriate. Multivariable linear regression was performed, with LDLc response to AP as the dependent variable. Those SNVs that showed an association with differences in the LDLc response to AP were included as independent variables, together with age, sex, basal values of BMI, cholesterol, LDLc, HDL cholesterol (HDLc), TG, and glucose.

## Results

*In silico* analysis performed with TRANSFAC®, MAPPER, and JASPER revealed that the following SNVs were located in regions that were of interest because of the presence of potential binding sites for transcription factors: rs2149041 (c.−3383C>G), rs2479406 (c.−2839A>C), rs17111503 (c.−2063A>G), rs2479408 (c.−1323C>G), and rs2479409 (c.−861A>G) in the *PCSK9* 5′ UTR, and rs17242346 (c.−2631G>A), rs17248720 (c.−2038C>T), and rs17249120 (c.−729G>A) in the *LDLR* 5′ UTR. By sequencing all fragments that contained these variants in the study subjects (treated and untreated), we identified a total of 23 variants: 13 in the *PCSK9* 5′ UTR, three in the *LDLR* 5′ UTR, and seven in the *LDLR* 3′ UTR. The frequencies of the minor allele ranged from 0.005 to 0.375 (S2 Table). However, to study the effects of genetics on total and LDL cholesterol response to AP, we considered only the treated subjects in further analyses.

The LDLc response to AP showed a nonskewed distribution. When subjects were classified according to their status as carriers of the minor allele of the studied SNVs, statistically significant differences (*p* < 0.05) were observed for c.−3383C>G and c.−2063A>G in the *PCSK9* 5′ UTR and rs14158 (c.*52G>A), rs2738465 (c.*504G>A), and rs2738466 (c.*773A>G) in the *LDLR* 3′ UTR. For all selected variants, a higher LDLc response to AP was observed for noncarriers of the minor alleles (Table 1). Total cholesterol response to AP showed a skewed distribution, but no statistically significant differences were found when comparing carriers vs noncarriers of minor alleles in the studied *PCSK9* and *LDLR* SNVs (data not shown).

**Table 1:** LDL cholesterol response to Armolipid Plus® in study subjects classified according to be carriers of the minor allele of the *PCSK9* and *LDLR* Variants.

Gene	Variants		LDLc response to AP		P
			Non-carriers	Carriers	
*PCSK9*	rs2149041	c.-3383C>G	-29.0 ± 15.11	-9.13 ± 28.03	0.008
	rs142236283	c.-3365G>A	-19.49 ± 23.02	-41.6	0.366
	rs79440992	c.-3363A>C	-17.76 ± 23.20	-34.10 ± 25.36	0.120
	rs140903350	c.-3082delAAGTT	-19.75 ± 24.87	-21,50 ± 17,93	0.870
	rs2479406	c.-2839A>C	-19,96 ± 24,54	-20,20 ± 20,14	0,984
	rs2495487	c.-2818A>T	-19,56 ± 24,35	-24,25 ± 20.77	0,713
	rs2479408	c.-1323C>G	-13,80 ± 22,24	-26,29 ± 24,93	0,090
	rs41294819	c.-925A>G	-17,66 ± 24,17	-29,07 ± 21,55	0.205
	rs2479409	c.-861A>G	-26,06 ± 16,87	-16,17 ± 26,98	0,184
	rs12096557	c.-1072G>A	-19,38 ± 24,20	-41,60	0,369
	rs17111503	c.-2063A>G	-29,4 ± 15,28	-12,15 ± 27,04	0,015
*LDLR*	rs17248720	c.-2038C>T	-17,88 ± 28,82	-25,33 ± 16,79	0.383
	rs36218923	c.-739A>T	-19,90 ± 24,15	-24,00	0,867
	rs17239120	c.-729G>A	-20,29 ± 24,07	-7.00	0.588
	rs17243004	c.*49G>A	-20,39 ± 24,35	-7.00	0.590
	rs14158	c.*52G>A	-25,58 ± 22,39	-10,81 ± 24,85	0,051
	rs3826810	c.*141G>A	-20,13 ± 24,14	-14,00	0,803
	rs2738464	c.*315C>G	-21,83 ± 21,72	-10,29 ± 33,49	0,245
	rs2738465	c.*504G>A	-25,58 ± 22,39	-10,81 ± 24,85	0,051
	rs1433099	c.*666A>T	-17,52 ± 24,65	-23,03 ± 23,83	0,462
	rs2738466	c.*773A>G	-25,58 ± 22,39	-10,81 ± 24,85	0,051

LDL colesterol response to Armolipid Plus® was expressed with mean ± SD. P-value was calculated by T-Student. AP: Armolipid Plus®; LDLc: LDL colesterol. P: p-value.

The effect of c.-3383C>G and c.-2063A>G changes in *PCKS9* have been analyzed with TRANSFAC®, MAPPER, and JASPER and a change in transcription factors pattern has been observed in both cases. Specifically, the *in silico* analysis has shown that the presence of the c.-3383G allele may implicate the abolition of a CD28RC element response.

Table 2 shows the results of multivariable linear regression analysis using the LDLc response to AP as the dependent variable. Variables that were independently associated with LDLc response to AP were status as carriers of *PCSK9* (c.−3383C>G) and *LDLR* (c.*52G>A) SNVs, with c.−3383C>G being the more significantly associated of the two variants. Together, these two minor alleles explained 20.5% of the variability in the LDLc response to AP. *PCSK9* c.−3383C>G accounted for 14.1% of the variability, while the remaining 6.4% was due to *LDLR* c.*52G>A. Gender, age, and basal clinical data were entered into this model, but no statistical significance was found.

**Table 2:** Genetic variables independently associated with LDLc response to Armpolipid Plus® by linear regression.

Variables	B	Standardized Coefficient (β)	p-value	Added Adjusted R^2^
PCSK9 c.-3383C>G (no/yes)	-17.725	6.717	0.012	0.141
LDLR c.*52G>A (no/yes)	-14.050	6.845	0.047	0.205

**Fig 1:**
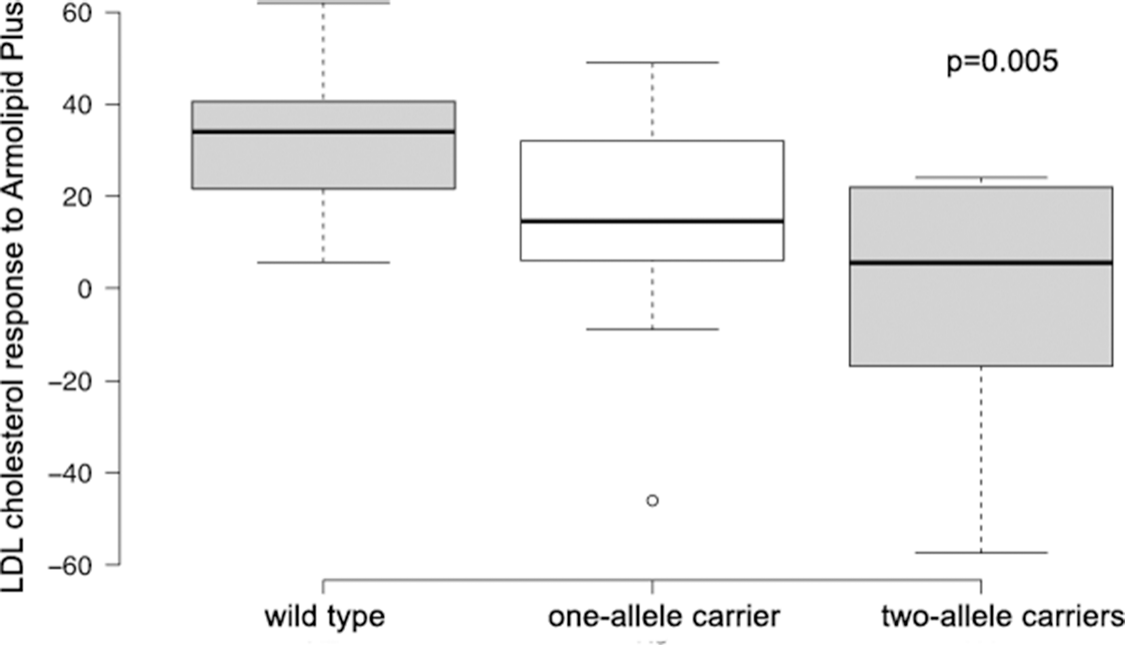
Study subjects classified according to status as carriers of c.−3383C>G *PCSK9* and c.*52G>A *LDLR* variants and response of LDL cholesterol levels to Armolipid Plus.

Fig 1 shows a boxplot of the haplotypes, with the variants c.−3383C>G in *PCSK9* and c.*52G>A in *LDLR*. Subjects were classified as wild type when they did not carry any minor allele, one-allele carriers, or two-allele carriers, and they were also classified according to their LDLc response to AP.

## Discussion

To the best of our knowledge, the present study was the first to analyze the effect of genetic variants in the 3′ UTR of *LDLR* and the 5′ UTR of *PCSK9* on response to the hypocholesterolemic effects of AP treatment. We confirmed that subjects with mild hypercholesterolemia showed a different plasma LDLc level response to AP if they were carriers of the common variants c.*52G>A in *LDLR* and c.−3383C>G in *PCSK9*.

Several studies have shown that the effects on plasma lipids of changes in dietary interventions differ among individuals, who are consequently classified as hyporesponders or hyper-responders [21–23]. The fact that extreme responses are reproducible in given individuals suggests that genetic factors may be involved [22, 24]. Treatment with AP was effective at reducing total cholesterol and LDLc levels in a previous randomized, placebo-controlled, crossover study of patients with moderate dyslipidemia. However, enormous variability was observed among individuals in their lipid-lowering response to AP treatment [13]. The mechanisms underlying the hypolipidemic effects of AP may be based on its main constituents. The lipid-lowering effects of berberine were first reported in Chinese patients with type IIa or IIb hyperlipidemia treated with berberine alone (500 mg twice per day) [17].

A limitation of the present study is that AP is a nutraceutical with several compounds; for example, red yeast rice extract is similar in structure to lovastatin, and it has been shown to have a cholesterol-lowering effect [25]. Although red yeast rice extract is a component of AP, its concentration compared to that of berberine is low [13]. Another limitation is that we only considered genetic variations in the 5′ and 3′ UTR of *LDLR* and the 5′ UTR of *PCSK9*. Other genetic variations could also influence the response to AP. Although the effect of the *PCSK9* 5’UTR SNVs c.-3383C>G and c.-2063A>G have been analyzed and differences in the transcription factors pattern have been observed, the specific effect of those in *PCSK9* regulation and its effect on AP response is not known. It has not been previously shown a relationship between CD28RC and *PCSK9*. To confirm our hypothesis further *in vitro* studies should be done such as EMSA and luciferase assays, unfortunately we have not been able to perform it due to budget restrictions.

Our hypothesis in the present study was that differences in the AP hypolipidemic response are mainly caused by genetic variants that affect the activity of berberine. We observed that mutations in the *LDLR* 3′ UTR and *PCSK9* 5′ UTR appeared to influence the AP response: carriers of c.−3383C>G and c.−2063A>G in *PCSK9* and c.*52G>A, c.*504G>A, and c.*773A>G in *LDLR* showed a lower response to AP treatment. Moreover, being a carrier of c.−3383C>G and c.*52G>A in *PCSK9* and *LDLR*, respectively, accounted for 20.5% of the variability in the LDLc response to AP. This may be due to differences in *LDLR* and *PCSK9* post-transcriptional and transcriptional regulation mediated by berberine.

*LDLR* expression is regulated at the post-transcriptional level by changes in mRNA stability [26], which is primarily controlled by regulatory sequences present in the 2.5 kb–long stretch of the 3′ UTR, where three mRNA destabilizing elements, called AU-rich elements (AREs), have been identified as responsible for the rapid turnover rate of LDLR mRNA. Previous investigations of these SNPs showed no differences in the allele distribution within subjects with or without coronary heart disease [26]. However, the effect of these variants on the response to AP, particularly berberine, has not been previously analyzed. We found a difference in the AP response in subjects who were carriers of c.*52G>A, c.*504G>A, and c.*773A>G. Variants c.*52G>A and c.*773A>G are located around the first and third AREs, respectively, and they are in linkage disequilibrium [27]. Only the c.*52G>A variant was included in the regression model, and it explained 6.4% of the response variability to AP. Recently, it was shown to have an effect on hnRNP D in the regulation of LDLR mRNA stability in berberine-treated mice [28].

Recently, berberine was also identified in HepG2 cells as a regulator of *PCSK9* transcriptional activity by inducing HNF1α via the ubiquitin proteasome system [29]. HNF1α is the principal form of HNF1 factors, and it stimulates *PCSK9* transcription. Whereas statins induce *PCSK9* transcription by enhancing the binding of SREBP2 to SRE-1, berberine decreases the cellular abundance of HNF1α and SREBP2, which results in reduced interaction of these two critical transactivators with their recognition sequences in the *PCSK9* promoter and leads to transcriptional repression. Carriers of minor alleles c.−3383C>G and c.−2063A>G, which are in the *PCSK9* 5′ UTR, showed a lower response to AP treatment. Moreover, the c.−3383C>G variant alone was responsible for 14.1% of the variability in the response to AP treatment. Moreover, we observed that being a carrier of one or two of the minor alleles *LDLR* c.*52G>A and *PCSK9* c.−3383C>G was associated with a lower response to AP.

In summary, three polymorphism in the *LDLR* 3′ UTR, c.*52G> A, c.*504 G>A, and c.*773A>G, and two in the *PCSK9* 5′ UTR, c.−3383C>G and c.−2063A>G, were associated with response to AP. The c.*52G>A *LDLR* variant, together with c.−3383C>G of *PCSK9*, could explain 20.5% of the variation in therapeutic response; carriers of one or more of these variants were more hyporesponsive to AP compared to those who were not carriers. These results could explain the differences in the response to berberine, and they suggest that analysis of these variants in individuals could be useful in providing people with personalized nutrition advice. Finally, based on our results and those of previous studies, we hypothesize that berberine has dual actions on LDLR metabolism, by prolonging the *LDLR* mRNA half-life as well as by directly increasing LDLR protein abundance through the blockage of PCSK9-mediated degradation. Thus, berberine and berberine-like compounds may be attractive therapeutic candidates for enhancing statin efficacy and should be investigated further in randomized clinical trials.

## Supporting Information

S1 Table Primers used for 5’UTR *PCSK9* and LDLR and 3’UTR LDLR sequencing. (DOCX)

S2 Table Variants found in the study cohort and frequency. (DOCX)

S3 Table Database used for statistical analysis. (XLSX)
