# Supplementary material for: Genetic Variants of LDLR and PCSK9 Associated with Variations in Response to Antihypercholesterolemic Effects of Armolipid Plus with Berberine
Source: PLoS One. 2016 Mar 25;11(3):e0150785. doi: 10.1371/journal.pone.0150785 (PMC4807809; doi:10.1371/journal.pone.0150785)
Supplement: S1 Table — (DOCX) [file pone.0150785.s001.docx]

**S1 Table.** Primers used for 5’UTR *PCSK9* and LDLR and 3’UTR LDLR sequencing.

| **Fragment** | **Forward** | **Reverse** |
| --- | --- | --- |
| PCSK9 5’UTR 01 | 5’ CCTAGCCTATTCTGATGGATTCCTC 3’ | 5’ TTAGAGCTGTGCCGACATAAGCC 3’ |
| PCSK9 5’UTR 02 | 5’ ATGGCTAGAAAAGCATTTTGAGGGACA 3’ | 5’ ACCACAAGTGCTTTCTGGGTCAACA 3’ |
| PCSK9 5’UTR 03 | 5’ TTACAGGCAACAGGAAGGAGAACCAC 3’ | 5’ ACCCTGCACACTGACCTTACCAA 3’ |
| LDLR 5’UTR 01 | 5’ TGTAGAGACAGGGTCCCACT 3’ | 5’ AGACGAGATTGCGCCACT 3’ |
| LDLR 5’UTR 02 | 5’ TTTCAATTTGACCTTGACACTGAGC 3’ | 5’ AGCCTCCTGAATAGCTAGGAC 3’ |
| LDLR 3’UTR | 5’ TGAGCAACAAAGCGAGAT 3’ | 5’ ATCACAGTAAGACATTCAGGCTA 3’ |
