# Supplementary material for: Genetic Variants of LDLR and PCSK9 Associated with Variations in Response to Antihypercholesterolemic Effects of Armolipid Plus with Berberine
Source: PLoS One. 2016 Mar 25;11(3):e0150785. doi: 10.1371/journal.pone.0150785 (PMC4807809; doi:10.1371/journal.pone.0150785)
Supplement: S2 Table — (DOCX) [file pone.0150785.s002.docx]

S2 Table. Variants found in the study cohort and frequency

| **GENE** | **SNP** | **Position** | **Major allele** | **Minor**  **allele** | **Minor allele Frequency** |
| --- | --- | --- | --- | --- | --- |
| **PCSK9** | rs2149041 | c.-3383 | C | G | 0.245 |
|  | rs142236283 | c.-3365 | G | A | 0.010 |
|  | rs79440992 | c.-3363 | A | C | 0.100 |
|  | rs140903350 | c.-3082 | AAGTT | delAAGTT | 0.055 |
|  | rs181789421 | c.-3054 | A | G | 0.005 |
|  | rs2479406 | c.-2839 | A | C | 0.110 |
|  | rs2495487 | c.-2818 | A | T | 0.075 |
|  | rs2479408 | c.-1323 | C | G | 0.286 |
|  | rs41294819 | c.-925 | A | G | 0.115 |
|  | rs2479409 | c.-861 | A | G | 0.375 |
|  | rs28385700 | c.-1110 | C | T | 0.015 |
|  | rs12096557 | c.-1072 | G | A | 0.050 |
|  | rs17111503 | c.-2063 | A | G | 0.315 |
| **LDLR** | rs17248720 | c.-2038 | C | T | 0.136 |
|  | rs36218923 | c.-739 | A | T | 0.115 |
|  | rs17249120 | c.-729 | G | A | 0.005 |
|  | rs17243004 | c.*49 | G | A | 0.005 |
|  | rs14158 | c.*52 | G | A | 0.235 |
|  | rs3826810 | c.*141 | G | A | 0.030 |
|  | rs2738464 | c.*315 | C | G | 0.085 |
|  | rs2738465 | c.*504 | G | A | 0.268 |
|  | rs1433099 | c.*666 | A | T | 0,263 |
|  | rs2738466 | c.*773 | A | G | 0.227 |
